# Bis(4-fluoro­benz­yl-κ*C*)bis­(3-methyl­sulfanyl-1,2,4-thia­diazole-5-thiol­ato-κ^2^ *N* ^4^,*S* ^5^)tin(IV)

**DOI:** 10.1107/S1600536811046654

**Published:** 2011-11-09

**Authors:** Ai-Xia Deng, Qian Xie, Mou-Yong Teng, Guo-Jia Fu

**Affiliations:** a College of Materials Science and Engineering, Liaocheng University, Shandong 252059, People’s Republic of China; b College of Chemistry and Chemical Engineering, Liaocheng University, Shandong 252059, People’s Republic of China

## Abstract

The mononuclear title molecule, [Sn(C_7_H_6_F)_2_(C_3_H_3_N_2_S_3_)_2_], has 2 symmetry. The Sn^IV^ atom, located on a twofold rotation axis, is in a skew trapezoidal–bipyramidal geometry, with the basal plane defined by two *S*,*N*-chelating 3-methyl­sulfanyl-1,2,4-thia­diazole-5-thiol­ate ligands. The apical positions are occupied by the C atoms of two 4-fluoro­benzyl groups.

## Related literature

For related structures, see: Ma *et al.* (2005 ▶); Zhang *et al.* (2005 ▶); Zhang *et al.* (2009 ▶).
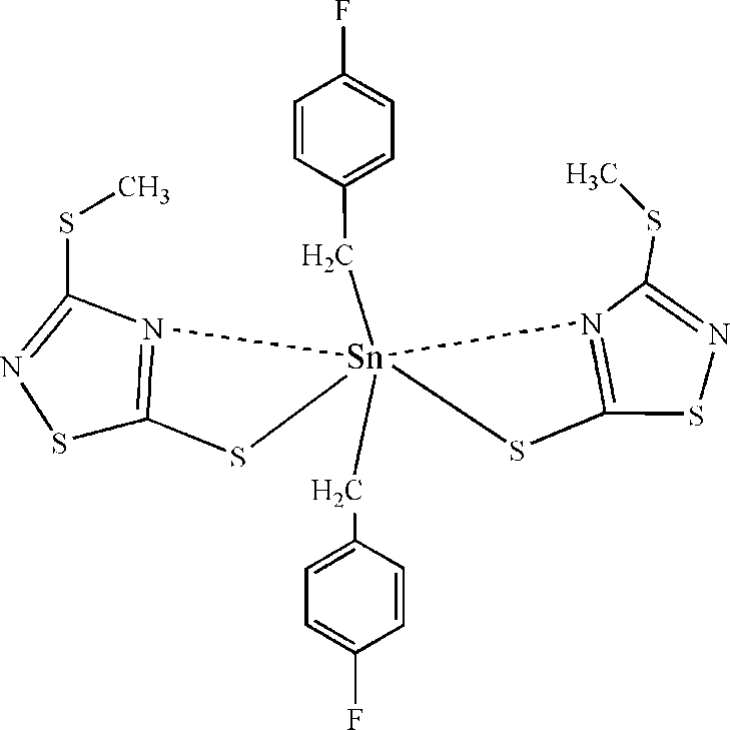


## Experimental

### 

#### Crystal data

[Sn(C_7_H_6_F)_2_(C_3_H_3_N_2_S_3_)_2_]
*M* *_r_* = 663.43
Monoclinic, 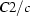

*a* = 13.9011 (14) Å
*b* = 17.769 (2) Å
*c* = 10.712 (1) Å
β = 104.081 (2)°
*V* = 2566.4 (5) Å^3^
*Z* = 4
Mo *K*α radiation
μ = 1.52 mm^−1^
*T* = 298 K
0.46 × 0.32 × 0.22 mm

#### Data collection

Bruker SMART 1000 CCD area-detector diffractometer
Absorption correction: multi-scan (*SADABS*; Bruker, 2001 ▶) *T*_min_ = 0.542, *T*_max_ = 0.732
6371 measured reflections
2269 independent reflections
2032 reflections with *I* > 2σ(*I*)
*R*_int_ = 0.025

#### Refinement

*R*[*F* ^2^ > 2σ(*F* ^2^)] = 0.025
*wR*(*F* ^2^) = 0.070
*S* = 1.15
2269 reflections
151 parameters
H-atom parameters constrained
Δρ_max_ = 0.39 e Å^−3^
Δρ_min_ = −0.55 e Å^−3^

### 

Data collection: *SMART* (Bruker, 2007 ▶); cell refinement: *SAINT* (Bruker, 2007 ▶); data reduction: *SAINT*; program(s) used to solve structure: *SHELXTL* (Sheldrick, 2008 ▶); program(s) used to refine structure: *SHELXTL*; molecular graphics: *SHELXTL*; software used to prepare material for publication: *SHELXTL* ▶).

## Supplementary Material

Crystal structure: contains datablock(s) I, global. DOI: 10.1107/S1600536811046654/xu5378sup1.cif

Structure factors: contains datablock(s) I. DOI: 10.1107/S1600536811046654/xu5378Isup2.hkl

Additional supplementary materials:  crystallographic information; 3D view; checkCIF report

## Figures and Tables

**Table 1:** Selected bond lengths (Å)

Sn1—C4	2.155 (3)
Sn1—S2	2.4703 (8)
Sn1—N1	2.913 (3)

**Fig. 1.**
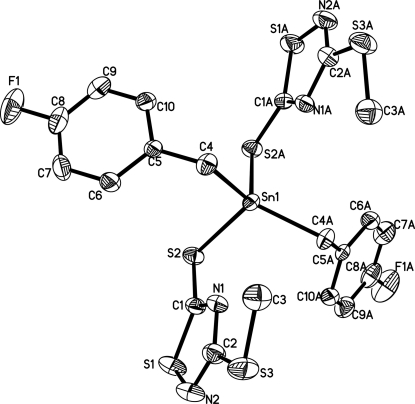
The molecular structure of the compound, showing 30% probability displacement ellipsoids.

**Fig. 2.**
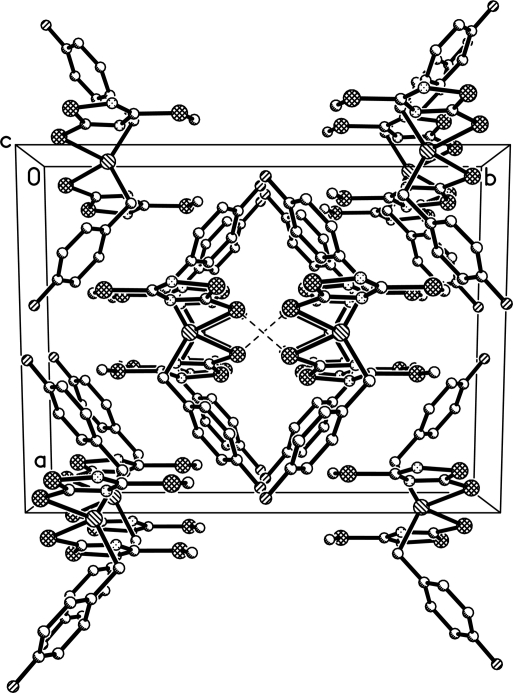
The unit cell of the title compound.

## supplementary crystallographic information

### Comment

In the title compound, from Fig.1, as far as the weak Sn—N interactions are concerned, the central Sn(IV) atom is situated in a skew-trapezoidal bipyramidal geometry, with the basal plane defined by two symmertrically chelating 3-methylmercapto-5-mercapto-1,2,4-thiadiazole ligands. The apical positions are occupied by two 4-fluorobenzyl groups. The coordination geometry of the Sn(IV) atom can also be described as distorted *trans* octahedral, with atoms N1, N1A, S2 and S2A occupying the equatorial positions, and atoms C4 and C4A occupying the axial positions. The molecular structure consists of a monomer with a hexa-coordinated Sn atom surrounded by two S atoms and two N atoms of the ligand, and two 4-fluorobenzyl groups.

The Sn—S bond distances and weak Sn—N bond lengths are 2.4703 (8)Å and 2.913 Å, respectively. The bite angles S2—Sn1—N1 and S2A—Sn1—N1A of title compound (59.12°) can be reconciled with a skew-trapezoidal bipyramidal geometry, although this geometry can also be considered as distorted *trans* octahedral. The structure of compound is close to those reported for a series of diorganotin(IV) 2-mercapto-4-methylpyrimidine derivatives (Ma *et al.*, 2005; Zhang *et al.*, 2005; Zhang *et al.* 2009). There is a good correspondence in their structure parameters: the Sn—S distances lie in the range 2.477–2.526Å and the Sn—N distances in the range 2.650–2.933 Å.

### Experimental

The 3-Methylmercapto-5-mercapto-1,2,4-thiadiazole (2 mmol) was added to the solution of ethanol 20 ml with sodium ethoxide (2 mmol), and the mixture was stirred for 30 minutes, then add Di(4-fluorobenzyl)dichlorotin(IV) (1 mmol) to the mixture, continuing the reaction for 12 h at 318k. After cooling down to room temperature, filtered it. The solvent of the filtrate was gradually removed by evaporation under vacuum until solid product was obtained. The solid was then recrystallized from ether-dichloromethane and colorless crystals suitable for X-ray diffraction were obtained (m.p. 410–412 K). Analysis, calculated for C_20_H_18_F_2_N_4_S_6_Sn: C 36.21, H 2.73, N 8.44, F 5.73; found: C 36.17, H 2.70, N 8.50, F 5.76%.

### Refinement

All H atoms were placed geometrically and treated as riding on their parent atoms, with methylene C—H distances of 0.97 Å, methyl and thiadiazole C—H distances of 0.96 Å.

### Crystal data

**Table:**

[Sn(C_7_H_6_F)_2_(C_3_H_3_N_2_S_3_)_2_]	*F*(000) = 1320
*M**_r_* = 663.43	*D*_x_ = 1.717 Mg m^−^^3^
Monoclinic, *C*2/*c*	Mo *K*α radiation, λ = 0.71073 Å
Hall symbol: -C 2yc	Cell parameters from 4420 reflections
*a* = 13.9011 (14) Å	θ = 2.5–28.2°
*b* = 17.769 (2) Å	µ = 1.52 mm^−^^1^
*c* = 10.712 (1) Å	*T* = 298 K
β = 104.081 (2)°	Block, colourless
*V* = 2566.4 (5) Å^3^	0.46 × 0.32 × 0.22 mm
*Z* = 4	

### Data collection

**Table:**

Bruker SMART 1000 CCD area-detector diffractometer	2269 independent reflections
Radiation source: fine-focus sealed tube	2032 reflections with *I* > 2σ(*I*)
graphite	*R*_int_ = 0.025
φ and ω scans	θ_max_ = 25.0°, θ_min_ = 1.9°
Absorption correction: multi-scan (*SADABS*; Bruker, 2001)	*h* = −16→16
*T*_min_ = 0.542, *T*_max_ = 0.732	*k* = −21→15
6371 measured reflections	*l* = −12→12

### Refinement

**Table:**

Refinement on *F*^2^	Primary atom site location: structure-invariant direct methods
Least-squares matrix: full	Secondary atom site location: difference Fourier map
*R*[*F*^2^ > 2σ(*F*^2^)] = 0.025	Hydrogen site location: inferred from neighbouring sites
*wR*(*F*^2^) = 0.070	H-atom parameters constrained
*S* = 1.15	*w* = 1/[σ^2^(*F*_o_^2^) + (0.0343*P*)^2^ + 2.4296*P*] where *P* = (*F*_o_^2^ + 2*F*_c_^2^)/3
2269 reflections	(Δ/σ)_max_ = 0.002
151 parameters	Δρ_max_ = 0.39 e Å^−^^3^
0 restraints	Δρ_min_ = −0.55 e Å^−^^3^

### Special details

**Table:**

Geometry. All e.s.d.'s (except the e.s.d. in the dihedral angle between two l.s. planes) are estimated using the full covariance matrix. The cell e.s.d.'s are taken into account individually in the estimation of e.s.d.'s in distances, angles and torsion angles; correlations between e.s.d.'s in cell parameters are only used when they are defined by crystal symmetry. An approximate (isotropic) treatment of cell e.s.d.'s is used for estimating e.s.d.'s involving l.s. planes.
Refinement. Refinement of *F*^2^ against ALL reflections. The weighted *R*-factor *wR* and goodness of fit *S* are based on *F*^2^, conventional *R*-factors *R* are based on *F*, with *F* set to zero for negative *F*^2^. The threshold expression of *F*^2^ > σ(*F*^2^) is used only for calculating *R*-factors(gt) *etc*. and is not relevant to the choice of reflections for refinement. *R*-factors based on *F*^2^ are statistically about twice as large as those based on *F*, and *R*- factors based on ALL data will be even larger.

### Fractional atomic coordinates and isotropic or equivalent isotropic displacement parameters (Å^2^)

**Table:**

	*x*	*y*	*z*	*U*_iso_*/*U*_eq_	
Sn1	0.5000	0.655810 (14)	0.2500	0.03453 (11)	
S1	0.62395 (8)	0.59377 (6)	−0.11579 (9)	0.0675 (3)	
S2	0.56268 (6)	0.55499 (4)	0.13199 (7)	0.0467 (2)	
S3	0.61389 (9)	0.82296 (6)	−0.08129 (10)	0.0711 (3)	
F1	0.9231 (2)	0.50566 (15)	0.6055 (3)	0.1095 (10)	
N1	0.58816 (19)	0.69118 (15)	0.0366 (2)	0.0437 (6)	
N2	0.6347 (3)	0.6831 (2)	−0.1563 (3)	0.0681 (9)	
C1	0.5912 (2)	0.61779 (18)	0.0233 (3)	0.0426 (7)	
C2	0.6129 (3)	0.7252 (2)	−0.0662 (3)	0.0530 (8)	
C3	0.6074 (3)	0.8515 (2)	0.0764 (4)	0.0667 (10)	
H3A	0.5470	0.8332	0.0936	0.100*	
H3B	0.6088	0.9055	0.0815	0.100*	
H3C	0.6631	0.8313	0.1387	0.100*	
C4	0.6259 (2)	0.71003 (17)	0.3750 (3)	0.0458 (7)	
H4A	0.6534	0.7464	0.3259	0.055*	
H4B	0.6041	0.7370	0.4420	0.055*	
C5	0.7043 (2)	0.65510 (15)	0.4355 (3)	0.0360 (6)	
C6	0.7802 (2)	0.6374 (2)	0.3788 (3)	0.0548 (8)	
H6	0.7821	0.6599	0.3011	0.066*	
C7	0.8538 (3)	0.5867 (3)	0.4351 (4)	0.0715 (12)	
H7	0.9050	0.5751	0.3964	0.086*	
C8	0.8491 (3)	0.5543 (2)	0.5482 (4)	0.0655 (10)	
C9	0.7749 (3)	0.5691 (2)	0.6068 (4)	0.0612 (9)	
H9	0.7729	0.5454	0.6836	0.073*	
C10	0.7031 (2)	0.6196 (2)	0.5500 (3)	0.0494 (8)	
H10	0.6522	0.6304	0.5897	0.059*	

### Atomic displacement parameters (Å^2^)

**Table:**

	*U*^11^	*U*^22^	*U*^33^	*U*^12^	*U*^13^	*U*^23^
Sn1	0.03463 (17)	0.03564 (17)	0.03042 (17)	0.000	0.00231 (11)	0.000
S1	0.0884 (7)	0.0725 (6)	0.0532 (5)	−0.0119 (5)	0.0395 (5)	−0.0207 (5)
S2	0.0602 (5)	0.0405 (4)	0.0424 (4)	−0.0011 (3)	0.0183 (4)	−0.0062 (3)
S3	0.0963 (8)	0.0655 (6)	0.0573 (6)	−0.0062 (5)	0.0300 (6)	0.0142 (5)
F1	0.0829 (18)	0.0850 (17)	0.133 (2)	0.0367 (14)	−0.0270 (16)	−0.0129 (17)
N1	0.0481 (15)	0.0487 (15)	0.0385 (14)	−0.0042 (12)	0.0190 (12)	−0.0013 (11)
N2	0.084 (2)	0.081 (2)	0.0493 (18)	−0.0115 (19)	0.0362 (17)	−0.0060 (16)
C1	0.0450 (17)	0.0514 (19)	0.0333 (16)	−0.0034 (14)	0.0133 (13)	−0.0074 (13)
C2	0.0525 (19)	0.062 (2)	0.0479 (19)	−0.0058 (16)	0.0188 (16)	0.0016 (16)
C3	0.084 (3)	0.051 (2)	0.067 (3)	−0.0025 (19)	0.021 (2)	0.0029 (17)
C4	0.0427 (17)	0.0433 (17)	0.0462 (18)	−0.0074 (13)	0.0006 (14)	−0.0081 (14)
C5	0.0308 (14)	0.0436 (16)	0.0310 (15)	−0.0080 (11)	0.0027 (11)	−0.0080 (12)
C6	0.0418 (18)	0.081 (2)	0.0431 (18)	−0.0046 (17)	0.0131 (15)	−0.0049 (17)
C7	0.041 (2)	0.097 (3)	0.075 (3)	0.0132 (19)	0.0120 (19)	−0.026 (2)
C8	0.051 (2)	0.056 (2)	0.076 (3)	0.0100 (17)	−0.0116 (19)	−0.0126 (19)
C9	0.059 (2)	0.066 (2)	0.050 (2)	−0.0059 (18)	−0.0021 (17)	0.0096 (17)
C10	0.0416 (17)	0.068 (2)	0.0384 (17)	−0.0042 (15)	0.0087 (14)	−0.0011 (15)

### Geometric parameters (Å, °)

**Table:**

Sn1—C4	2.155 (3)	C3—H3B	0.9600
Sn1—C4^i^	2.155 (3)	C3—H3C	0.9600
Sn1—S2^i^	2.4703 (8)	C4—C5	1.489 (4)
Sn1—S2	2.4703 (8)	C4—H4A	0.9700
Sn1—N1	2.913 (3)	C4—H4B	0.9700
S1—N2	1.663 (4)	C5—C6	1.376 (4)
S1—C1	1.715 (3)	C5—C10	1.383 (4)
S2—C1	1.727 (3)	C6—C7	1.387 (5)
S3—C2	1.745 (4)	C6—H6	0.9300
S3—C3	1.786 (4)	C7—C8	1.356 (6)
F1—C8	1.370 (4)	C7—H7	0.9300
N1—C1	1.314 (4)	C8—C9	1.357 (6)
N1—C2	1.371 (4)	C9—C10	1.370 (5)
N2—C2	1.313 (4)	C9—H9	0.9300
C3—H3A	0.9600	C10—H10	0.9300
			
C4—Sn1—C4^i^	126.88 (17)	C5—C4—H4A	109.2
C4—Sn1—S2^i^	109.90 (9)	Sn1—C4—H4A	109.2
C4^i^—Sn1—S2^i^	107.95 (9)	C5—C4—H4B	109.2
C4—Sn1—S2	107.95 (9)	Sn1—C4—H4B	109.2
C4^i^—Sn1—S2	109.90 (9)	H4A—C4—H4B	107.9
S2^i^—Sn1—S2	87.03 (4)	C6—C5—C10	117.6 (3)
N2—S1—C1	92.81 (15)	C6—C5—C4	121.1 (3)
C1—S2—Sn1	92.51 (10)	C10—C5—C4	121.3 (3)
C2—S3—C3	101.08 (16)	C5—C6—C7	121.3 (3)
C1—N1—C2	109.2 (2)	C5—C6—H6	119.3
C2—N2—S1	107.4 (2)	C7—C6—H6	119.3
N1—C1—S1	111.3 (2)	C8—C7—C6	118.3 (3)
N1—C1—S2	123.4 (2)	C8—C7—H7	120.9
S1—C1—S2	125.31 (19)	C6—C7—H7	120.9
N2—C2—N1	119.2 (3)	C7—C8—C9	122.5 (3)
N2—C2—S3	119.3 (3)	C7—C8—F1	118.3 (4)
N1—C2—S3	121.5 (2)	C9—C8—F1	119.1 (4)
S3—C3—H3A	109.5	C8—C9—C10	118.4 (3)
S3—C3—H3B	109.5	C8—C9—H9	120.8
H3A—C3—H3B	109.5	C10—C9—H9	120.8
S3—C3—H3C	109.5	C9—C10—C5	121.9 (3)
H3A—C3—H3C	109.5	C9—C10—H10	119.1
H3B—C3—H3C	109.5	C5—C10—H10	119.1
C5—C4—Sn1	112.03 (19)		

Symmetry codes: (i) −*x*+1, *y*, −*z*+1/2.
